# Postnatal Outcomes and Risk Factors for In-Hospital Mortality among Asphyxiated Newborns in a Low-Resource Hospital Setting: Experience from North-Central Nigeria

**DOI:** 10.5334/aogh.2884

**Published:** 2020-06-18

**Authors:** Taofik Oluwaseun Ogunkunle, Halim Odiachi, Jerry Rueben Chuma, Surajudeen Oyeleke Bello, Abdulazeez Imam

**Affiliations:** 1 Department of Paediatrics, Dalhatu Araf Specialist Hospital, Lafia, Nasarawa State, NG; 2 Department of Vaccines and Immunity, Medical Research Council Unit The Gambia at London school of Hygiene and Tropical Medicine, Atlantic Boulevard, Fajara, GM

## Abstract

**Background:**

Birth asphyxia accounts for a third of global newborn deaths and 95 percent of these occur in low-resource settings. A key to reducing asphyxia-related deaths in these settings is improving care of these newborns and this requires an understanding of factors associated with adverse outcomes.

**Objectives:**

In this study, we report outcomes and risk factors for mortality among newborn infants with birth asphyxia admitted to a typical low-resource hospital setting.

**Methods:**

We prospectively followed up 191 asphyxiated newborn infants admitted to a referral tertiary hospital in North-central Nigeria. At baseline, care-givers completed a structured questionnaire. Using univariable analysis, we compared baseline characteristics between participants who died and those who survived till discharge. We also fitted a multivariable logistic regression model to identify risk factors for mortality among the cohort.

**Results:**

Majority (60.7%) of the study participants presented to the hospital within the first six hours of life. Despite this, mortality among the cohort was 14.7% with a third dying within the first 24 hours of admission. The presence of respiratory distress at admission increased the risk for mortality (AOR = 3.73, 95% CI 1.22 to 11.35) while higher participant weight at admission decreased the risk (AOR = 0.11, 95% CI 0.03 to 0.40). Intrapartum factors such as duration of labour and maternal age, although significant on univariable analysis, were not significant on multivariable analysis.

**Conclusions:**

Hospital mortality among newborns with birth asphyxia is high in North-central Nigeria and majority of deaths occur during acute care. Respiratory distress at presentation and admission weights were identified as key risk factors for asphyxia mortality. Intrapartum factors on the other hand might have indirect effects on mortality through an increased risk for neonatal complications.

## Background

Globally, the last three decades have witnessed considerable decline in under five mortality, but neonatal mortality has not mirrored this feat [1]. Birth asphyxia now accounts for about a quarter of newborn deaths and is associated with significant morbidity among survivors, who suffer disabling motor and cognitive deficits [1,2]. There is currently significant global variation in morbidity and mortality among neonates with asphyxia, as greater than 95% of all asphyxia-related deaths and impairment in these group occur in low resource settings [3].

A prominent target of the third sustainable development goal is the global reduction of under-five mortality [4]. A key strategy to attaining this is provision of better care for sick newborn infants, particularly in resource-poor settings and this requires a better understanding of factors that are associated with adverse health outcomes in these settings.

Nigeria has the greatest burden of birth asphyxia in sub-Saharan Africa [5]. In 2018, the country was one of two countries which accounted for a third of global under five deaths and in the same period recorded about 267,000 newborn infant deaths, with asphyxia accounting for roughly 30% of these [1]. Additionally, asphyxia accounts for between 18 and 30% of newborn admissions in Nigeria [6,7,8]. In-hospital mortality among these infants varies significantly across reported local studies and in some instances is greater than 25% [8,9]. As such, the country represents a typical resource-constrained high burden setting for birth asphyxia.

In this study, we prospectively followed up and determined the factors associated with in-patient mortality among neonates who were admitted with asphyxia to a typical low-resource referral newborn health centre in North-central Nigeria. Such data is critical for identifying potential targets for intervention and also to improve care of these infants in this setting and similar low-resource settings.

## Subject and Methods

### Study design and setting

This was a prospective cohort study which followed up newborn infants admitted to the Special Care Baby Unit (SCBU) of the Dalhatu Araf Specialist Hospital (DASH). DASH is the only referral tertiary health facility providing neonatal services in Nasarawa state. The state is located in North-central Nigeria and has a population of about 1.8 million [10]. The DASH SCBU is a 35 bed nursery served by a paediatric consultant, a senior resident, four medical officers and nurses working on shifts. It is equipped with functional incubators, radiant warmers, oxygen delivery units, suction units and bag-mask devices. Like a typical low-resource newborn facility, it has no provisional for blood gas analysis and does not have any neonatal cooling systems for managing asphyxiated infants. There are also no conventional facilities to provide neonatal ventilator support and the SCBU relies on improvised bubble continuous positive airway pressure (CPAP) devices. These are constructed using neonatal nasal prongs with tubing, an oxygen source and a calibrated plastic container which contains a column of water [11,12].

### Study population

The study population were newly admitted asphyxiated newborn infants at the SCBU at DASH. Perinatal asphyxia was defined using pre-set clinical criteria as described by other studies in low-resource settings, where blood gas analysis is not routinely performed [13,14]. This criteria was based on a fifth minute Apgar score ≤6 and/or the presence of delayed or absent crying at birth and/or sentinel events (e.g. prolong/obstructed labour, pre-eclampsia/eclampsia, intrapartum foetal distress, placental abruption etc.), prolonged resuscitation and/or any acute features of neurologic dysfunction (hypotonia, coma, convulsions, apnoea or depressed reflexes) not explained by other obvious factors.

We excluded neonates with congenital abnormalities, central nervous system malformations, those with maternal history of receiving opioids or other depressant medications as this could have confounded our clinical diagnosis of perinatal asphyxia.

### Data collection

Following care-giver informed consent, we prospectively recruited consecutive study participants who fulfilled our pre-set clinical criteria from January to December, 2019. We administered structured questionnaires to collect participant socio-demographic details, clinical history, maternal medical and pregnancy history and observed for signs of encephalopathy while determining severity using the Sarnat and Sarnat classification [15]. Participant gestational ages were also determined using the Ballard scoring system. All participants had their admission weights measured, a baseline blood sugar performed and they were followed up until our outcome of interest (death or discharge). During follow-up, we recorded whether or not convulsion occurred and determined length of hospital stay for the participants.

### Ethical considerations

Ethical approval for our study was obtained from the Research Ethics Committee of Dalhatu Araf Specialist Hospital (Reference number – DASH/L/ADM/0340) and informed consent was obtained from parents or guardians of all enrolled participants prior to any study procedures.

### Data analysis

We provided summary statistics for participant data, describing frequencies and percentages and providing means and standard deviation for normally distributed data and median with inter-quartile range for non-normally distributed data. Baseline participant characteristics were compared between newborn infants with asphyxia who survived till discharge and those who died using chi-square to compare proportions, t-tests for normally distributed data and Man-Whitney U test to compare medians. We considered differences in both groups to be statistically significant when p-values were less than 0.05.

To adjust for possible confounding, we performed multivariable analysis using logistic regression analysis, determining odd ratios and their corresponding 95% confidence intervals. All statistical analyses were performed using STATA version 13 (Stata Corp. 2013. *Stata Statistical Software: Release 13*. College Station, TX: Stata Corp LP)

## Results

We recruited one hundred and ninety one (191) newborns with birth asphyxia. Our cohort comprised 130 (68.1%) males and 61 females (31.9%). Median age at hospital presentation (±Inter-quartile range) of the cohort was 3 (±19) hours. Majority (80.6%) were discharged, while mortality among the cohort was 14.7%. Two participants (1.1%) discharged against medical advice and 7 (3.7%) absconded from care.

**Figure 1:**
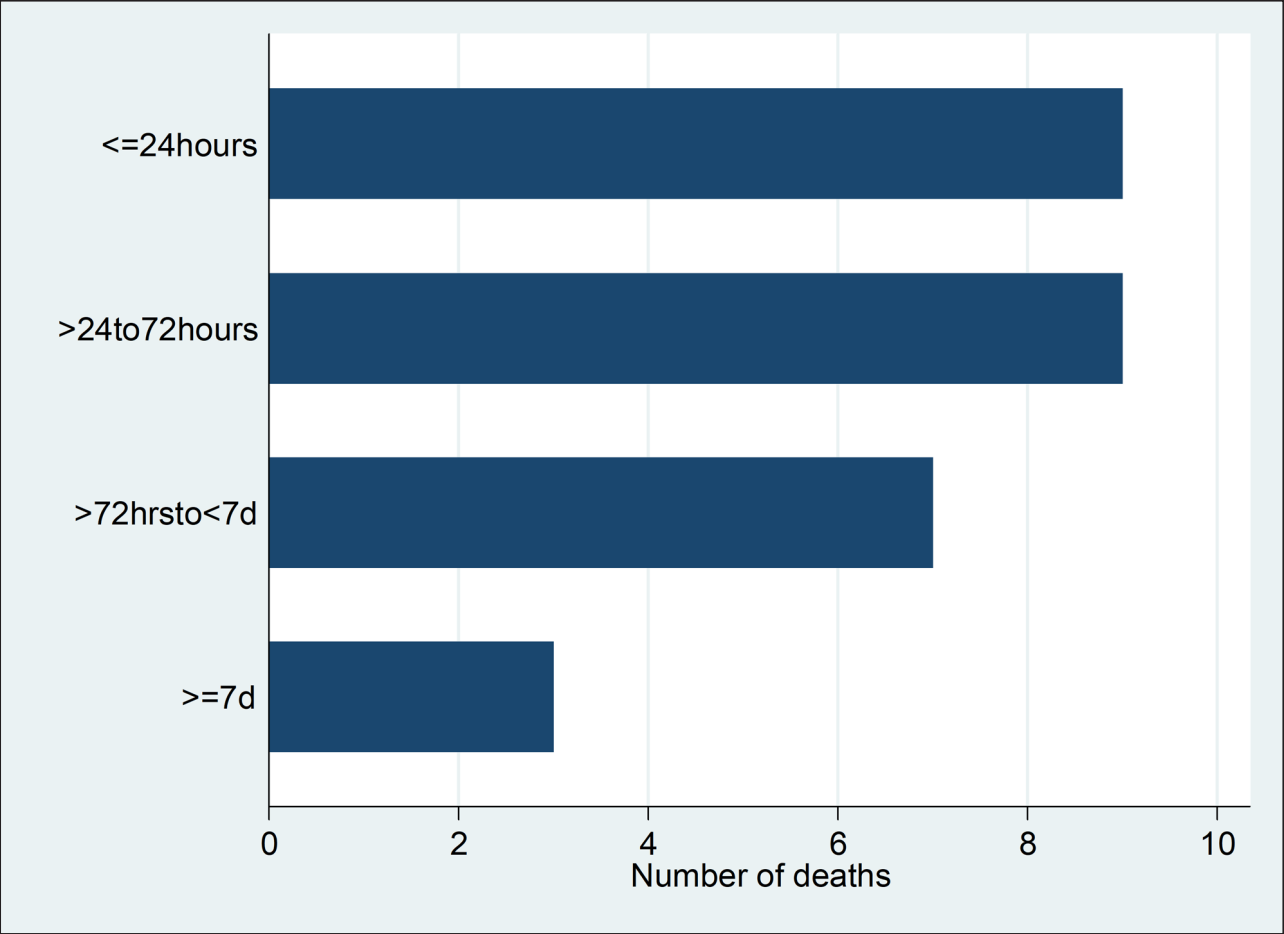
Horizontal bar graph showing number of deaths across time.

There were 28 deaths in the cohort. About one-third of deaths occurred within 24 hours of presentation, while about two-thirds died within 72 hours of admission. The least number of deaths occurred after 7days (Figure 1).

### Participant socio-demographic and baseline clinical features

Table 1 shows baseline clinical features and socio-demographics for our cohort. Majority (60.7%) of participants presented early to the health facility, presenting within 6 hours of life. Additionally, age at clinical presentation did not differ significantly between participant who died and those who survived (Table 1, p-value – 0.90). Both groups did not also differ significantly in terms of gender, admitting random blood sugar levels, gestational age proportions and place of birth. Study participants who died however had significantly lower mean admission weights (p-value – 0.01) and a larger proportion of this group had Hypoxic Ischaemic Encephalopathy stages two and three when compared to those who survived (p-value – <0.001). They also had significantly larger proportion of babies with poor suck, respiratory distress and altered consciousness (Table 1).

**Table 1:** Sociodemographic and baseline clinical parameters of individual study participant (n = 191).

Variable	Participants who died (%) n = 28	Survived till discharge (%) n = 163	P-value	Total (%) n = 191

**Age at clinical presentation (hours)**				
<1	6 (21.4)	37 (22.7)	0.90	43 (22.5)
1 to 6	10 (35.7)	63 (38.7)		73 (38.2)
7 to 24	7 (25.0)	29 (17.8)		36 (18.9)
25 to 48	2 (7.1)	18 (11.0)		20 (10.5)
>48	3 (10.7)	16 (9.8)		19 (10.0)
**Sex**				
Male	18 (64.3)	112 (68.7)	0.64	130 (68.1)
Female	10 (35.7)	51 (31.3)		61 (31.9)
**Gestational age category**				
Term	19 (67.9)	133 (81.6)	0.13	152 (79.6)
Preterm	7 (25.0)	27 (16.6)		34 (17.8)
Post-term	2 (7.1)	3 (1.8)		5 (2.6)
**Place of birth**				
Home	21 (12.9)	6 (21.4)	0.77	27 (14.1)
Private hospital	7 (4.3)	1 (3.6)		8 (4.2)
Traditional birth attendant clinic	2 (1.2)	0 (0.0)		2 (1.1)
Primary Health Care centre	38 (23.3)	4 (14.3)		42 (22.0)
Secondary level health facility	15 (9.2)	3 (10.7)		18 (9.4)
Tertiary health care centre	80 (49.1)	14 (50.0)		94 (49.2)
**Mean ± SD admission weight (Kg)**	2.4 ± 0.7	2.7 ± 0.6	0.01*	2.7 ± 0.8
**Median ± IQR admission RBS (mmol/L)**	2.95 ± 2.5	3.6 ± 2.5	0.13	3.6 ± 2.6
**HIE**				
No HIE	6 (21.4)	55 (33.7)	<0.001*	61 (31.9)
Presumed HIE	1 (3.6)	13 (8.0)		14 (7.3)
HIE 1	8 (28.6)	80 (49.1)		88 (46.1)
HIE 2	12 (42.9)	14 (8.6)		26 (13.6)
HIE 3	1 (3.6)	1 (0.6)		2 (1.1)
**Delayed cry**				
Yes	28 (100.0)	162 (99.4)	0.68	190 (99.5)
No	0 (0.0)	1 (0.6)		1 (0.5)
**Pallor**				
Present	5 (17.9)	12 (7.4)	0.07	17 (8.9)
Absent	23 (82.1)	151 (92.6)		174 (91.1)
**Cyanosis**				
Present	3 (10.7)	15 (9.2)	0.80	18 (9.4)
Absent	25 (89.3)	148 (90.8)		173 (90.6)
**Respiratory distress**				
Present	20 (71.4)	70 (42.9)	0.005*	90 (47.1)
Absent	8 (28.6)	93 (57.1)		101 (52.9)
**Depressed Moro reflexes**				
Present	21 (75.0)	95 (58.3)	0.09	116 (60.7)
Absent	7 (25.0)	68 (41.7)		75 (39.3)
**Altered consciousness**				
Present	12 (42.9)	13 (8.0)	< 0.001*	25 (13.1)
Absent	16 (57.1)	150 (92.0)		166 (86.9)
**Convulsions**				
Present	14 (50.0)	74 (45.4)	0.65	88 (46.1)
Absent	14 (50.0)	89 (54.6)		103 (53.9)
**Poor suck**				
Present	22 (78.6)	92 (56.4)	0.027*	114 (59.7)
Absent	6 (21.4)	71 (43.6)		77 (40.3)

**HIE –** Hypoxic Ischaemic Encephalopathy; **IQR** – Interquartile Range; **SD** – Standard deviation; * **–** Significant at P < 0.05.

### Participant maternal and family characteristics

Table 2 shows maternal and family characteristics of our study participants. The proportion of teenage mothers was significantly higher among participants who died when compared to those who survived (14.3% versus 3.7%, p-value = 0.02). Both groups also differed significantly in terms of maternal education level and duration spent in labour. Mothers of participants who died were on average less educated and had longer duration of labour than those who survived till discharge. Other variables such as maternal parity, maternal employment, place of residence, family type, history of eclampsia, maternal history of hypertension, mode of delivery, antenatal care attendance, and place of antenatal care did not differ significantly between the groups.

**Table 2:** Matenal and family characteristics of individual study participant (n = 191).

Variable	Participants who died (%) n = 28	Survived till discharge (%) n = 163	P-value	Total (%) n = 191

**Maternal age**				
<18	4 (14.3)	6 (3.7)	0.02*	10 (5.2)
≥18	24 (85.7)	157 (96.3)		181 (94.8)
**Parity**				
Primiparous	14 (50.0)	66 (40.5)	0.35	80 (41.9)
Multiparous	14 (50.0)	97 (59.5)		111 (58.1)
**Maternal education**				
Tertiary	1 (3.6)	40 (24.5)	0.045*	41 (21.5)
Secondary	10 (35.7)	37 (22.7)		47 (24.6)
Primary	8 (28.6)	29 (17.8)		37 (19.4)
No formal education	9 (32.1)	57 (35.20		66 (34.6)
**Paternal education**				
Tertiary	6 (21.4)	64 (39.2)	0.23	70 (36.7)
Secondary	11 (39.3)	39 (23.9)		50 (26.2)
Primary	3 (10.7)	19 (11.7)		22 (11.5)
No formal education	8 (28.6)	41 (25.2)		49 (25.7)
**Maternal Employment**				
Yes	20 (71.4)	88 (54.0)	0.09	108 (56.5)
No	8 (28.6)	75 (46.0)		83 (43.5)
**Place of residence**				
Within hospital locality	18 (64.3)	121 (74.2)	0.28	139 (72.8)
Outside hospital locality	10 (35.7)	42 (25.8)		52 (27.2)
**Family type**				
Monogamous	21 (75.0)	134 (82.2)	0.37	155 (81.2)
Polygamous	7 (25.0)	29 (17.8)		36 (18.9)
**Attended antenatal Care**				
Yes	28 (100.0)	158 (96.9)	0.35	186 (97.4)
No	0 (0.0)	5 (3.1)		5 (2.6)
**ANC location**				
Tertiary	0 (0.0)	13 (8.0)	0.19	13 (6.8)
Secondary	18 (64.3)	82 (50.3)		100 (52.4)
Primary Health Care	2 (7.1)	6 (3.7)		8 (4.2)
Private Care	3 (10.7)	10 (6.1)		13 (6.8)
None	5 (17.9)	52 (31.9)		57 (29.8)
**Duration of labour (hours)**				
Median ± IQR	17 ± 17	12 ± 10	0.004*	13 ± 10
**Mode of delivery**				
SVD	109 (66.9)	16 (57.1)	0.53	125 (65.5)
Emergency CS	47 (28.8)	11 (39.3)		58 (30.4)
Elective CS	4 (2.5)	0 (0.0)		4 (2.1)
Instrumental delivery	3 (1.8)	1 (3.6)		4 (2.1)
**Maternal hypertension**				
Pregnancy induced HTN	4 (14.2)	22 (13.5)	0.70	26 (13.6)
Chronic HTN	0 (0.0)	4 (2.5)		4 (2.1)
No	24 (85.7)	137 (84.1)		161 (84.3)
**Eclampsia**				
Yes	0 (0.0)	4 (2.5)	0.40	4 (2.1)
No	28 (100.0)	159 (97.6)		187 (97.9)

**ANC –** Antenatal care**; CS –** caesarean section; **HTN –** Hypertension; **HIE –** Hypoxic Ischaemic Encephalopathy; **IQR** – Interquartile Range; **SD** – Standard deviation; **SVD –** Spontaneous Vertex Delivery; * **–** Significant at P < 0.05.

### Risk factors for mortality

Table 3 shows odds ratios for mortality among the recruited cohort. For each additional kilogram a participant had at admission, the odds for in-hospital mortality decreased by 89% (Table 3, AOR = 0.11, 95% CI 0.03 to 0.40). Additionally, the presence of respiratory distress at clinical presentation was associated with a 3.7 times increased odds for mortality (Table 3, AOR = 3.73, 95% CI 1.22 to 11.35). Presence of altered consciousness, poor suck, hypoxic ischaemic encephalopathy staging, maternal age, maternal education level and duration of labour were not associated with in-patient mortality on multivariable analysis (Table 3).

**Table 3:** Crude odds ratios, adjusted odds ratios and 95% confidence intervals of risk factors for mortality in the recruited cohort (n =191).

Variable	Crude odds ratios and 95% confidence interval	Adjusted odds ratios and 95% confidence interval^&^

**Admission weight (Kg)**	0.41 (0.20 to 0.82)	0.11 (0.03 to 0.40)
**Altered consciousness**		
Present	8.65 (3.38 to 22.13)	4.59 (0.43 to 49.03)
Absent	1	1
**Poor suck**		
Present	2.83 (1.09 to 7.35)	1.40 (0.40 to 4.91)
Absent	1	1
**Respiratory distress**		
Present	3.32 (1.38 to 7.98)	3.73 (1.22 to 11.35)
Absent	1	1
**HIE**		
No HIE	1	1
Presumed HIE	9.17 (0.51 to 166.11)	1.66 (0.06 to 43.65)
HIE 1	0.71 (0.08 to 6.37)	0.69 (0.05 to 8.92)
HIE 2	0.92 (0.30 to 2.79)	2.37 (0.47 to 12.04)
HIE 3	7.86 (2.50 to 24.62)	12.43 (0.81 to 191.59)
**Maternal age**		
<18	4.36 (1.15 to 16.59)	4.02 (0.55 to 29.42)
≥18	1	1
**Maternal education**		
Tertiary	0.16 (0.02 to 1.30)	0.35 (0.03 to 3.55)
Secondary	1.71 (0.64 to 4.61)	1.61 (0.44 to 5.96)
Primary	1.75 (0.61 to 5.00)	1.35 (0.35 to 5.23)
No formal education	1	1
**Duration of labour (hours)**	1.02 (1.00 to 1.05)	1.01 (0.98 to 1.05)

**HIE –** Hypoxic Ischaemic Encephalopathy.

## Discussion

Our study investigated post-natal outcomes and risks factors for mortality among asphyxiated newborn infants admitted to a typical low-resource neonatal care setting in North-central Nigeria. We found that one in seven newborn infants admitted with asphyxia died while in hospital, and 3.7% absconded from medical care. Respiratory distress at presentation increased the odds for mortality while higher admission weight was associated with reduced odds for mortality. Antenatal care attendance and intrapartum factors such as duration of labour and teenage motherhood were not significant risk factors for mortality on multivariable analysis.

The in-hospital case fatality rate for asphyxia in this study was 14.7% and about a third of these newborn infants died within 24 hours of admission, and this was despite early presentation to hospital. These asphyxia case fatality rates are similar to those previously described in studies from other low-resource settings [16,17]. In contrast, mortality prevalence was significantly higher in a previous North-western Nigerian study were rates of 25.5% were described [8]. These differences might be due to variations in rates of maternal co-morbidities such as eclampsia which affect baseline clinical severity of asphyxiated newborns. In this study, only 2.1% of mothers presented with eclampsia, while the previous study documented 23.9% of participant mothers as having eclampsia when they presented. Additionally, about two-thirds of our cohort presented within 6 hours of life and median age at presentation in this study was 3 hours. Despite this early presentation, a third of deaths occurred on the first day of presentation. High mortality among asphyxiated newborns in the first 24 hours has also been reported in some other similar setting to ours [18]. These findings have important implications, as they signify challenges with acute care of asphyxiated newborn infants. Acute care of sick newborn requires stabilising, monitoring and early recognition of clinically deteriorating newborn infants. Similar to many neonatal units in poor resource settings, patient to health staff ratios are high in our setting and we do not have continuous monitoring devices for many of our infants. Such factors would affect acute sick newborn care.

A second adverse outcome described in our study was absconsion from medical care which was 3.7 percent. Absconding from care occurs when parents of admitted infants unanimously discharge their wards from health facilities without the knowledge of health professionals or the hospital authorities. Like many low-resource settings, local health expenditure is primarily out-of-pocket in Nigeria and many newborn infant come from low-income families, as such absconding from hospital is not uncommon. In one study in a similar setting to ours, it was estimated that median family expenditure on care for in-patient newborn infants with asphyxia was almost 10 percent of the average annual family income [19]. Such costs are frequently impossible for families to bear. Additionally, aside care costs, a relative absence of strong child protection services and local punitive laws further perpetuates this.

The current study also showed that asphyxiated newborn infants who presented in respiratory distress were more than three times likely to die than those without breathing problems. Such neonates often require positive pressure ventilation either in the form of mechanical ventilation or continuous positive airway pressure (CPAP) ventilation, both of which are not present in the current study location and many low-resource settings. In our setting, we currently provide respiratory support using locally improvised bubble CPAP devices that have been shown to be effective and safe among neonates and older children in similar settings to ours [12,20,21]. Sustained oxygen supply is critical for appropriate functioning of these devices and this currently represents a challenge in our setting, where oxygen is paid for primarily out-of-pocket by care-givers. In many instances, even when such therapies are started, sustenance is difficult leading to significant challenges at managing these sick newborns.

Higher admission weight was shown to decrease the odds for mortality among asphyxiated newborn infants. The importance of birth weight for newborn infant survival has also been described in other studies [22,23]. Infant birth weight is strongly correlated with maternal nutrition during pregnancy and the occurrence of adverse birth weight outcomes have been linked to co-existing maternal illnesses and teenage pregnancy [24,25]. While we found no relationships between antenatal care (ANC) attendance and risk for mortality among our cohort, we believe the key to an impactful ANC is not just in attendance, but lies in the quality of care provided. In many poor-resource settings, challenges exist in the quality of ANC care provided [5]. As such, ensuring adequate nutrition in pregnancy, conducting quality antenatal care and promoting girl child education to reduce teenage marriage and pregnancy, all of which primarily prevent birth asphyxia, might also improve the chances of asphyxiated newborn survival through improvements in birth weight in low-resource settings [5].

Additionally, on univariable analysis, we found intrapartum factors, notably, duration of labour, teenage motherhood and maternal education status to be associated with asphyxia mortality. These risk factors however lost statistical significance on further multivariable analysis. While all three factors are well recognised risks for asphyxia, their loss of statistical significance on multivariable analysis for mortality, suggests they might indirectly exert their effects on mortality through other proxy factors. Our multivariable models consisted of both intrapartum and clinical neonatal factors. While intrapartum risk factors lost statistical significance, neonatal clinical factors maintained statistical significance. This suggests, for example, an increase in duration of labour (an intrapartum factor) in itself might not translate to an increased risks for mortality among asphyxiated newborns in low-resource settings, particularly in the absence of neonatal complications such as respiratory distress.

## Strengths and limitations

We have conducted a prospective study and have used multivariable logistic regression to address possible confounders for risk estimates. This study thus improves on previous studies that have not adjusted for the effect of confounding or have been retrospective case analysis [16]. The prospective nature of our study means we were able to recruit and collect data on all our participants using pre-set clinical criteria and hence minimising selection bias. We have also described additional risk factors associated with in-hospital mortality of asphyxiated newborn infants in low-resource settings that have not been identified by previous studies [13,14].

Although we could not use arterial blood gas to define cases of asphyxia because this test is not performed at the study location, we have used strict clinical case definition criteria that have been used in other studies from similar low-resource settings [13,14]. Additionally, we provided some data on study participants’ intrapartum care, but were unable to provide data on cadre of the individuals conducting these deliveries and use of oxytocin during labour, both of which might have been risk factors for asphyxia mortality.

## Conclusion

Mortality remains high among neonates admitted with perinatal asphyxia in North-central Nigeria and a significant proportion of these babies die during acute care. Key risk factors identified for mortality where clinical neonatal factors such as respiratory distress and admission weight.

While Intrapartum factors are well known risk factors for asphyxia, our findings suggest these factors might have indirect effects on mortality through increased occurrence of neonatal complications. We did not find any association between antenatal care and asphyxia mortality, raising possible concerns about the quality of antenatal care provided in our setting

## Data Accessibility Statement

The datasets that were used for analysis and preparation of this manuscript are available from the corresponding author upon reasonable request.

## Abbreviations

ANC – Antenatal Care

CPAP – Continuous Positive Airway Pressure

DASH – Dalhatu Araf Specialist Hospital

HIE – Hypoxic Ischaemic Encephalopathy

SCBU – Special Care Baby Unit
